# Death by Irritation

**Published:** 1841-11

**Authors:** 


*Death by Irritation.*—From the Boston Atlas we learn that Henry Cooledge, of Framingham, Mass., recently died under the following singular circumstances. Having shaved the face of his dead father, he soon after used the same razor in shaving himself. Although the father had died a natural death, and nothing remarkable had been observed in the manner of his decease, the face and head of the son began to swell almost immediately after having finished the operation of shaving, and he was himself soon a corpse. The absorption of virus from the dead body, if introduced on the edge of the instrument into the system of the son, seems not to have been there sufficiently long to have circulated, and his death is to be imputed, therefore, according to the writer in the Atlas, to irritation.—*Boston Med. and Surg. Jour.*

